# Sports sponsorship, gambling bans, and regulatory compliance: a systematic review

**DOI:** 10.3389/fspor.2026.1900549

**Published:** 2026-08-06

**Authors:** Erik Obiol-Anaya, Iván Vargas-Chaves, José Rolando Cardenas-Gonzales

**Affiliations:** 1 School of Law, San Martín de Porres University, Lima, Peru; 2 School of Law, Nueva Granada Military University, Bogotá, Colombia; 3 School of Law, Complutense University of Madrid, Madrid, Spain

**Keywords:** brand recall for sponsor, compliance, gambling marketing, globalization of sport, sports marketing

## Abstract

**Introduction:**

This systematic review evaluates the impact of sports sponsorship bans and betting restrictions on marketing practices, regulatory compliance, and consumer behavior following global market liberalization.

**Methods:**

Following PRISMA guidelines and the SPICE framework, we searched five major academic databases for peer-reviewed literature from 2022 onwards, synthesizing 29 eligible studies evaluated via the Mixed Methods Appraisal Tool (MMAT).

**Results:**

Gambling sponsorship has expanded rapidly in professional sports over two decades. Comprehensive statutory bans significantly reduce marketing exposure and new gambling registrations, whereas partial or voluntary self-regulatory restrictions lead to marketing displacement and circumvention tactics such as sub-branding.

**Discussion:**

Partial and voluntary restrictions are insufficient to protect public health and sports integrity. Comprehensive statutory prohibitions covering primary and derivative proxy brands, supported by strong enforcement and alternative funding for sports organizations, are essential for effective policy implementation.

## 1 Introduction

In recent decades, professional sports organizations and the gambling industry have become increasingly interconnected. Sponsorships, advertising, and broadcasting partnerships now provide significant financial support for athlete salaries, stadiums, and grassroots initiatives (1). Meanwhile, the liberalization of sports betting in regions such as the United Kingdom, Australia, and parts of Europe has led to greater scrutiny of this relationship. This development raises important questions about sports integrity, consumer protection, and the effectiveness of current regulations (2).

Gambling-related harm has significant and well-documented social and public health consequences. Gambling disorder, recognized as a behavioral addiction, is associated with financial hardship, mental health decline, relationship breakdown, and increased suicide rates (3). Children and young people are particularly vulnerable to marketing, as early exposure to gambling advertising can normalize betting and accelerate the development of gambling problems (4). Sports sponsorship is a powerful marketing channel that embeds gambling brands within the emotional context of athletic competition and team loyalty, strongly influencing attitudes and behaviors among young audiences (5).

In response to these concerns, governments and regulatory bodies have introduced various policies to limit gambling sponsorship in sport. These include complete statutory bans, as in Italy and Spain, partial restrictions on advertising during certain times or in specific media, and industry-led self-regulatory codes based on voluntary compliance (6). This range of approaches enables comparison of regulatory effectiveness in achieving public health goals. However, evidence on the efficacy of these restrictions remains fragmented, as most studies focus on single jurisdictions, specific sports, or particular marketing channels (7).

A significant research gap remains in systematically synthesizing evidence on how gambling bans and betting restrictions affect sponsorship prevalence and content, as well as the behavior of gambling operators, sports organizations, and consumers. While some narrative and scoping reviews have examined the extent of gambling marketing in sport (8), few have rigorously compared the effectiveness of different regulatory mechanisms in achieving compliance and reducing harm. Research on regulatory circumvention, such as sub-branding, exploiting loopholes, or shifting advertising to unregulated areas, is limited but concerning (9). Policymakers need to understand these adaptive strategies to develop robust and effective regulations. Additionally, the perspectives of key stakeholders, including fans, athletes, and sports administrators, on the ethical aspects of gambling sponsorship are underexplored, yet are essential for understanding the social license for these practices (2).

This systematic review provides a comprehensive, evidence-based synthesis to inform policy development in this important public health area. As sports betting markets expand globally, particularly through digital and mobile platforms, the risk of gambling-related harm is expected to increase. Policymakers require clear evidence on which regulatory interventions are most effective, for whom, and under what circumstances, to allocate resources efficiently and protect vulnerable groups. This review meets this need by systematically gathering and analyzing empirical research from public health, sports management, marketing, and regulatory fields. Its contribution is to compare regulatory models, focus on implementation and compliance, and synthesize stakeholder perspectives that are often isolated in individual studies.

This review also addresses a key gap by examining how the strictness of gambling sponsorship bans influences the prevalence and nature of marketing exposure. Although stricter regulations are expected to reduce sponsorship, operator responses may limit these effects. For example, a ban on jersey sponsorship may lead operators to focus on stadium naming rights or digital advertising, shifting rather than reducing exposure (10). By systematically documenting these displacement effects and circumvention tactics across jurisdictions, this review provides important insights for designing comprehensive regulations that minimize unintended outcomes.

## 2 Methodology

### 2.1 Review protocol

We conducted a systematic literature review following the guidelines established by the PRISMA (Preferred Reporting Items for Systematic Reviews and Meta-Analyses) statement (11). The review protocol was designed *a priori* to ensure transparency, reproducibility, and rigor in the study selection and synthesis processes. The research question was formulated using the SPICE framework, which guided the development of appropriate search terms and inclusion criteria. Additionally, to ensure full transparency and compliance with standard reporting practices for systematic reviews, a completed PRISMA checklist is provided as an appendix to this manuscript.

The literature search was executed across five bibliographic databases selected for their multidisciplinary coverage and relevance to the research domain. Scopus provided a comprehensive interdisciplinary index across health sciences, social sciences, and sports management. Web of Science was selected for its curated content and citation tracking, which helped identify key studies in gambling regulation and sponsorship. ScienceDirect contributed peer-reviewed journals in the social and behavioral sciences, where much of the relevant literature is found. SpringerLink offered a wide range of books and journals in sports science, public health, and regulatory policy. Google Scholar was used to access grey literature, official reports, and studies from smaller regional journals, ensuring a thorough and inclusive search.

The search strategy was developed iteratively through pilot testing and refinement. The final search string combined controlled vocabulary (e.g., Medical Subject Headings) and free-text terms, adapted to the syntax of each database.

For Scopus, the following Boolean string was used: TITLE-ABS-KEY [(“sport sponsorship” OR “sports sponsorship” OR “sponsorship in sport” OR “betting sponsorship” OR “gambling sponsorship”) AND (“gambling ban” OR “gambling bans” OR “betting restriction” OR “betting restrictions” OR “gambling advertising ban” OR “gambling advertising bans” OR “regulatory compliance” OR “gambling regulation” OR “gambling regulations”)). In Web of Science, we employed: TS = (“sport sponsorship” OR “sports sponsorship”) AND (“gambling ban*” OR “betting restriction*” OR “regulatory compliance” OR “gambling regulation*”).

For ScienceDirect and SpringerLink, the simplified string “sports sponsorship” AND (“gambling bans” OR “betting restrictions” OR “regulatory compliance”) was used, as these platforms have more limited Boolean capabilities. Google Scholar was searched using the keywords: “sports sponsorship” “gambling bans” OR “betting restrictions” OR “gambling advertising bans” OR “regulatory compliance” “gambling regulation”.

All searches were performed in January 2025, and the results were limited to publications from 2022 onwards. While seminal regulatory frameworks, such as the UK's voluntary “whistle-to-whistle” ban (2019) and Spain's Royal Decree 958/2020, were enacted earlier, the 2022 threshold was deliberately chosen to capture the empirical evaluations of these policies post-implementation. Regulatory impact assessment typically requires a latency period to observe stabilized effects on sponsorship arrangements, consumer behavior, and industry adaptation. Therefore, restricting the search to the most recent years ensures the synthesis relies on mature, retrospective empirical data rather than early predictive studies. The total number of records retrieved from all databases was 373.

### 2.2 Analytical dimensions for synthesis and synthesis procedure

To organize the diverse evidence identified in our search, we developed a set of analytical dimensions aligned with the main themes of the research question and the varied methodologies of the included studies. These dimensions provided a structured framework for data extraction and synthesis, enabling systematic comparison across studies addressing the sports sponsorship and gambling regulation nexus.

The first dimension examines the exposure, content, and prevalence of gambling marketing in sport, using quantitative content analysis, observational audits, or longitudinal tracking to document the frequency, placement, and nature of sponsorship messages across sports, leagues, and media platforms. This establishes a baseline for assessing regulatory interventions. The second dimension evaluates the effectiveness and impact of gambling advertising and sponsorship restrictions. Studies here use quasi-experimental designs, natural experiments, or comparative policy analyses to determine whether regulatory frameworks, such as bans or self-regulation, reduce marketing exposure, consumer recall, or gambling-related harm. This directly informs policy effectiveness. The third dimension explores stakeholder perceptions and ethical considerations, drawing on qualitative methods like interviews, focus groups, or surveys to understand the attitudes and reasoning of fans, athletes, administrators, and policymakers regarding gambling sponsorship in sport.

This provides essential context for regulation implementation and public response. A final, residual dimension includes studies that do not fit the primary categories but offer relevant insights, such as legal analyses, economic impact assessments, or methodological papers. This framework guided data extraction and thematic synthesis, ensuring comprehensive coverage of the research question.

### 2.3 Inclusion and exclusion criteria

Inclusion and exclusion criteria were defined to ensure all studies in the review were directly relevant to the research question and met high methodological standards. Eligible studies addressed the intersection of sports sponsorship with gambling bans, betting restrictions, or regulatory compliance. Accepted study types included empirical research using quantitative, qualitative, or mixed methods; systematic reviews and meta-analyses; legal or policy analyses; and case studies of specific jurisdictions, sports, or sponsorship arrangements. Studies had to be published in peer-reviewed journals or as official reports from recognized governmental or regulatory bodies, such as national gambling commissions, public health agencies, or sports governing bodies. Only studies with full text available in English were included.

Each study was required to explicitly address at least one of the following: the impact of sponsorship bans on sports organizations, the effectiveness of betting restrictions in sports, or compliance mechanisms for gambling advertising and sponsorship regulations. Studies were excluded if they focused solely on general gambling behavior or addiction without reference to sports sponsorship or betting restrictions. Opinion pieces, editorials, commentaries, conference abstracts, and non-peer-reviewed preprints were excluded due to their preliminary or non-empirical nature. Studies on sponsorship of non-gambling products, such as alcohol or soft drinks, were not included.

Descriptive studies lacking a clear methodological framework, such as marketing brochures or industry white papers without empirical data, were excluded. Studies without accessible full text after reasonable retrieval efforts were omitted. Publications duplicating data or findings from another included study, such as multiple papers from the same dataset without new analysis, were also excluded.

### 2.4 Study selection process

The study selection followed four stages in accordance with PRISMA guidelines. First, database searches identified 373 records. After removing 161 duplicates through software and manual checks, 212 unique records advanced to screening. Two independent reviewers screened titles and abstracts against eligibility criteria, resolving disagreements through discussion or a third reviewer. This stage excluded 130 records, mainly for focusing on general gambling, non-sport sponsorship, or lacking regulatory compliance. In the second stage, full-text reports were sought for the remaining 82 records.

Despite efforts, 10 could not be retrieved due to access issues or non-responsiveness. The third stage involved assessing the 72 available full texts for methodology, outcomes, and relevance. Forty-three reports were excluded for not addressing sports sponsorship and gambling regulation (12), lacking methodological rigor or data (13), being non-peer-reviewed opinion pieces or abstracts (10), or duplicating data (6). The final sample included 29 studies suitable for synthesis, as shown in the PRISMA flowchart in Supplementary Figure S1. Several limitations should be noted.

The reliance on English-language publications may have excluded relevant research from non-English-speaking regions, particularly in Asia and South America. Excluding grey literature and industry reports improved methodological rigor but may have omitted valuable observational data on operator compliance and circumvention. The focus on recent publications (2022 onwards) ensured currency but may have excluded important historical studies. Finally, variability in methodological quality, especially between quantitative and qualitative studies, complicates synthesis and limits definitive conclusions.

In accordance with PRISMA guidelines, a risk of bias assessment was conducted for the included studies. Two independent reviewers evaluated the methodological quality of the empirical studies using the Mixed Methods Appraisal Tool (MMAT), which accommodates the diverse range of study designs (quantitative, qualitative, and mixed methods) included in this review. Discrepancies were resolved through consensus. While the overall quality of the included studies was high, some descriptive and observational studies exhibited a moderate risk of bias due to self-reported measures or the absence of control groups. These limitations were carefully considered during the data synthesis and the interpretation of the regulatory impacts.

## 3 Results

### 3.1 Research trends

The annual distribution of publications shows a marked increase in academic interest in sports sponsorship as it relates to gambling bans, betting restrictions, and regulatory compliance. As shown in Supplementary Figure S2, the number of studies rose from three in 2022 to eleven in 2025, more than tripling over four years. This growth reflects a focused scholarly response to policy developments, rather than a general rise in academic publishing. Notably, the surge in 2023 and 2024, with seven and eight publications respectively, aligns with major regulatory changes, such as the Gambling Act Review in the United Kingdom and stricter sponsorship rules in Belgium and the Netherlands.

This timing suggests that researchers are actively evaluating the effects of new regulations and contributing to policy discussions. The peak in 2025 may indicate a maturing research field, with more advanced comparative and evaluative studies emerging. The apparent drop to three publications in 2026 likely results from the search being conducted in January 2025, before most 2026 studies were indexed. Overall, this pattern highlights the timeliness and policy relevance of the current review, which captures a period of significant scholarly engagement with ongoing regulatory reforms.

The recent concentration of publications reflects a shift in research focus. Studies from 2022 to 2023 primarily documented the prevalence and nature of gambling sponsorship in sport, often using content analysis of television broadcasts or social media to establish baseline data. In contrast, research from 2024 to 2025 increasingly uses quasi-experimental designs, natural experiments, and comparative policy analyses to assess the effectiveness of regulatory interventions. This methodological shift advances the field from descriptive studies to causal inference and evidence-based policy recommendations. The geographic scope has also expanded.

While early research focused on the United Kingdom and Australia, recent studies examine regulatory contexts in Italy, Spain, Belgium, and the Netherlands, offering a broader evidence base for understanding different regulatory models. This diversification is essential for identifying factors that affect the success of sponsorship restrictions, such as enforcement strength, cultural attitudes toward gambling, and the structure of sports leagues. These trends show a field evolving in both methodological rigor and geographic reach, making this systematic review a timely synthesis of a dynamic and policy-relevant body of evidence.

### 3.2 Overview of included studies

Supplementary Table S1 summarizes the main characteristics of the included studies. Extracted data cover study identification, design, setting, population, sponsorship or advertising type, regulatory context, measured outcomes, and key findings, where applicable.

The included studies differed in study design, regulatory context, and outcome measures. These variations are important for interpreting the review's findings and identifying potential sources of heterogeneity. The characteristics table offers a structured summary of the studies and forms the basis for the following narrative or quantitative synthesis.

### 3.3 Exposure, content, and prevalence of gambling marketing in sport

Research consistently shows that gambling marketing in sports is frequent, diverse, and often aggressively targeted across various jurisdictions, sports, and media channels. Evidence from content analyses, audits, and surveys demonstrates that gambling marketing is now deeply integrated into both professional sports and esports. This widespread presence has significant implications for audience exposure, especially among vulnerable groups such as young people.

A retrospective study of shirt sponsorships in ten major football leagues from 2000 to 2022 (14) highlights this trend. Of 4,452 sponsorship records, gambling sponsors on team jerseys rose from 1.7% in 2000 to 16.3% in 2022, while alcohol-related sponsorships fell from 6.2% to 1.0%. Although most sponsorships (81.8%) were classified as “other,” gambling became the largest single category of potentially harmful sponsorship, surpassing alcohol and unhealthy food.

This shift reflects a deliberate strategy by the gambling industry to align with popular sports and leverage fans’ emotional connections. The trend is also evident in esports: a review of the top 20 teams in Dota 2, CS:GO, and League of Legends (15) found that half of the Dota 2 and CS:GO world championship teams in 2021 were sponsored by gambling companies. These teams had a combined social media following of over 25 million, providing direct access to a young, tech-savvy audience. The lack of gambling sponsors in League of Legends, despite related teams in other games, suggests a strategic response to different platform policies and audience sensitivities.

The frequency and nature of gambling advertising during live sports broadcasts are concerning, especially given the high viewership among children and adolescents. A content analysis of 32 Czech First Football League matches (16) recorded 1,824 gambling advertisements, averaging 57 per match and over six hours of airtime. Sports betting made up 85% of these ads, but online casino and lottery promotions were also common. In Ireland, a study of 65 live televised male sporting events (17) found gambling ads in 75.4% of games, ranking them as the seventh most advertised product.

Notably, gambling ads were most frequent during football matches and often aired before the adult television watershed, exposing children to direct marketing. In the UK, despite a voluntary “whistle-to-whistle” ban, gambling ads remain common during live sports. During the men's 2020 Euro soccer tournament, 113 gambling adverts were recorded, averaging 4.5 per match (18). Similarly, ITV's coverage of the 2022 Qatar World Cup featured 156 gambling adverts across 30 matches, with 80.8% shown pre-match to encourage immediate betting (19). These adverts mainly promoted financial inducements, such as sign-up offers and boosted odds, which are particularly effective at stimulating gambling behavior (18).

Gambling marketing now uses sophisticated strategies such as celebrity endorsements, promotional inducements, and social media engagement, often with little emphasis on responsible gambling information. A mixed-methods analysis of NFL broadcasts in the United States (12) found that celebrity appearances in sports betting ads increased the perceived trustworthiness of operators and reduced viewers’ risk perception, more so than monetary incentives. This suggests that trusted figures can normalize gambling and lower perceived risks. A UK survey of 18–24-year-olds during the 2022 FIFA World Cup (20) showed almost universal recall of sports betting ads, with higher-risk gamblers recalling more ads than lower-risk gamblers.

Most advertising was recalled from social media (10–14 ads per week), followed by internet banners and television, while less than half remembered seeing responsible gambling messages. An experimental study (21) found that sign-up and bonus bet inducements had a stronger influence on increasing betting and riskier gambling among young people than other offers, and those with gambling problems were more likely to believe these incentives could motivate riskier behavior. These findings show that gambling marketing is carefully designed to maximize appeal and behavioral impact, especially among vulnerable groups.

Analysis of social media and digital channels reveals further exposure and regulatory challenges. A study of gambling ads during NHL and NBA matches in Ontario, Canada, and related social media posts (22), found 4,119 gambling messages across both platforms. While none of the 3,537 television ads violated Ontario advertising standards, nearly half (48.5%) of the 582 X/Twitter ads were classified as content marketing that concealed their promotional nature, breaching standards. These posts generated over 5.6 million views and mainly targeted males aged 25–34, a group with high sports engagement. This highlights a key regulatory challenge: distinguishing legitimate content from covert advertising in the fast-paced environment of social media.

A more subtle form of exposure has emerged as gambling companies use sub-branding strategies to bypass sponsorship bans. In Belgium, where strict regulations limit gambling sponsorship, operators have created sub-brands in sectors like sports media (23, 24). These sub-brands, such as “Circus Daily,” retain clear links to their parent gambling brands through shared names, typography, and colors. A qualitative survey of 200 Belgian sports fans (23) found that participants easily recalled these sub-brands and associated them with their parent brands, mainly through semantic and categorical cues. This supports the idea that even indirect exposure to sub-brands can trigger associations with gambling companies, undermining the intent of sponsorship bans. The regulatory implication is clear: bans on explicit gambling brands may be ineffective if operators can shift to closely related sub-brands that maintain brand awareness.

To provide a structured overview of the evidence base, Supplementary Table S2 summarizes the key characteristics of studies focused on exposure, content, and prevalence.

While evidence shows that gambling marketing is widespread, some studies have compared sponsorship exposure or examined specific advertising channels. For example, research tracking gambling marketing during a Premier League match weekend in the UK (25) found it remains common across live broadcasts, sports news, radio, and social media, demonstrating multi-channel saturation. The impact of a complete ban is also notable. A study of the Spanish Royal Decree 958/2020, which imposed comprehensive restrictions on gambling marketing and sponsorship (13, 26), found a permanent decrease in new gambling accounts and total money bet, along with significant reductions in advertising, bonuses, and sponsorship spending. However, the link between marketing and behavior weakened for most channels except bonuses, where the impact intensified. This suggests that even with effective bans, the remaining marketing, especially financial inducements, can still have a strong influence.

### 3.4 Effectiveness and impact of gambling advertising and sponsorship restrictions

Assessing the practical effectiveness of gambling advertising and sponsorship restrictions is central to policy discussions on regulating gambling marketing in sport. The studies reviewed present a complex and sometimes conflicting view of regulatory effectiveness, showing that outcomes depend heavily on the design, scope, enforcement, and industry adaptation. Overall, comprehensive, legally enforced bans are more effective at reducing marketing exposure and related gambling behaviors than partial or voluntary measures, though all frameworks remain vulnerable to circumvention.

The strongest evidence for strict regulation comes from studies of the Spanish Royal Decree 958/2020, which imposed broad limits on gambling advertising, bonuses, and sponsorships (13, 26). Using SARIMA models and official data, researchers found a lasting decrease in key gambling behaviors after the decree, including 263,000 fewer new gambling accounts and a €216 million drop in total bets (13). Marketing expenditures also fell: advertising by €20 million, bonuses by €2.6 million, and sponsorship by €5.3 million (13). This demonstrates that legal bans on sponsorship reduce financial ties between gambling companies and sports organizations, lowering brand visibility in sports. However, the regulation did not significantly change the behavior of existing gamblers (13), suggesting that sponsorship bans are more effective at preventing new gambling than altering established habits.

A follow-up analysis showed that, before the ban, each €1 spent on bonuses and sponsorship led to gamblers depositing €1.6 and €4, respectively. After the decree, most links between marketing spend and gambling behavior weakened, except for bonuses, where the effect increased (26). This highlights an unintended consequence: restricting most marketing channels can make the remaining ones, like bonuses, more influential. Regulatory frameworks should therefore address all forms of promotion, not just advertising and sponsorship.

In contrast to the Spanish approach, evidence on partial and voluntary restrictions, such as the UK's industry-led “whistle-to-whistle” (W2W) ban, shows limited and inconsistent effectiveness. The W2W ban, intended to reduce children's exposure to gambling ads during live sports, was evaluated through several quasi-experimental studies (27–29). These studies found a significant reduction of about 2.3 gambling ads per live football broadcast, mainly during half-time (28), indicating some benefit in specific contexts. However, the ban led to increased advertising during live horse racing and pre-match football segments, both outside the ban's scope (27–29).

On average, three gambling ads per program remained after the ban (28). This displacement effect and continued advertising presence raise concerns about the effectiveness of voluntary, self-regulatory measures. Researchers concluded that stronger statutory regulations are likely needed to address gambling advertising harms effectively (29). The W2W ban illustrates how the industry can adapt to and exploit gaps in partial or voluntary restrictions.

Research also assesses the effectiveness of restrictions at local and organizational levels, revealing a gap between policy intent and implementation. An audit of 139 local governments in Western Australia found that only 6% had any policy restricting the promotion of harmful products, including gambling, and just 23 had at least one policy limiting unhealthy signage or sponsorship (30). This lack of local policy infrastructure leaves community sports vulnerable to gambling marketing, especially as elite leagues reduce such sponsorships (31). In elite sport, regulatory responses vary widely. For example, the English Premier League will ban gambling shirt-front sponsorships by 2026, but this will not cover shirt-sleeve sponsors or pitch-side hoardings, which are the most visible forms of in-game marketing. In contrast, Italy and Spain have adopted comprehensive bans on gambling sponsorship and in-game marketing, which are considered stronger public health measures (32). These comparisons show that the effectiveness of a ban depends on its scope; partial bans allow significant marketing exposure to persist.

A sophisticated form of regulatory circumvention is the use of sub-brands, which undermines sponsorship restrictions even in countries with strict laws. In Belgium, where a comprehensive ban was enacted, gambling operators launched sub-brands like “Circus Daily” that operate in sectors such as sports media but retain strong visual and semantic links to their parent gambling companies (23, 24). Surveys of Belgian football fans (*n* = 200) showed that fans easily recognized these sub-brands and associated them with the parent companies, mainly through branding cues like logos, typography, and colors (23, 24). This association allows sub-brand sponsorships to act as proxies for banned brands, defeating the legislation's intent. Fans reported frustration and viewed these tactics as deliberate exploitation of legal loopholes, sometimes considering them even more inappropriate than direct sponsorship (24). This evidence indicates that effective sponsorship bans must also cover related or derivative brands that could serve as marketing proxies.

To provide a structured synthesis of the regulatory evaluations, Supplementary Table S3 details the key characteristics of the studies focusing on effectiveness and impact.

The effectiveness of restrictions also depends on the nature of the sponsorship. An experimental study found that consumers saw a poor fit between gambling companies and soccer teams and judged such sponsorships as morally inappropriate (33). This moral judgment negatively affected both the sponsoring brand and the team. These findings suggest that consumer resistance to gambling sponsorship provides a public health rationale for bans beyond reducing exposure, as bans also reinforce and legitimize public disapproval of these partnerships.

### 3.5 Stakeholder perceptions and ethical considerations of gambling sponsorship

The ethical implications of gambling sponsorship in sport are central but often underexplored in the literature. Studies reviewed here show that stakeholders—including fans, community sports leaders, and policymakers—face complex moral tensions, weighing the financial needs of sport against the known harms of gambling. These views differ based on stakeholder roles, sponsor types, sport levels, and regulatory environments. Multiple qualitative studies consistently find that stakeholders see a fundamental conflict between the values of health, fair play, and integrity in sport and the profit-driven, risk-oriented values of the gambling industry.

Focus group research with Australian sporting fans (34) highlighted this “contradiction.” Participants recognized the paradox of linking sport's positive, healthy image with gambling, a product known to cause harm. This moral dissonance was a major concern, especially regarding children's exposure to such marketing. Of the “unhealthy commodities” studied (unhealthy food, alcohol, and gambling), gambling was seen as having the greatest health impact, prompting stronger support for restrictions. An experimental study further found that consumers, especially those without personal gambling issues, viewed gambling sponsorship as morally inappropriate and the sponsor as insincere (33). A poor fit between the gambling company and the soccer team intensified negative moral judgments, harming both the sponsor and the team. These findings suggest that public ethical standards act as a check on these partnerships, and that bans on gambling sponsorship align with consumer values.

The ethical debate becomes more complex when financial viability is considered. Although fans and community leaders may oppose gambling sponsorship on ethical grounds, the significant revenue it generates creates strong pressure to accept it. In-depth interviews with senior leaders in Australian community sporting organizations found that decisions about gambling sponsorship were “influenced by a range of perceived risks and benefits” (35). Participants weighed value alignment with sponsors against the need for financial stability, with most agreeing that gambling and sport should not be linked. Notably, not all gambling sponsorship was seen as equally harmful. Local sponsorships, such as from neighborhood pubs with a few poker machines, were viewed as less problematic than those from large online wagering companies (35). This hierarchy of perceived harm complicates regulatory efforts to establish consistent rules.

This tension between ethical values and financial needs was further examined in a related study of the same Australian cohort (31). The study found that sporting organizations face “ethical dilemmas when considering gambling sponsorships, weighing values, reputation, and financial viability.” Some leaders said they would reject gambling sponsorship for ethical reasons, but acknowledged that this stance is often only possible for wealthier clubs. Clubs with fewer resources were seen as “more vulnerable to taking up gambling sponsorship” (31), creating a situation where the most financially disadvantaged communities are most likely to accept funding from a harmful industry. The study also warned that as elite clubs move away from gambling sponsorship due to regulation or public pressure, gambling companies may increasingly “target community sport” (31), shifting harm to more vulnerable sectors. This highlights the need for policies that not only ban gambling sponsorship but also provide alternative, non-harmful funding for community sport, so that ethical decisions do not disproportionately burden disadvantaged groups.

The tactic of regulatory circumvention through sub-branding has generated strong negative ethical reactions from fans. Qualitative surveys of Belgian football fans found that many expressed “frustration and a sense of betrayal” when they realized gambling companies were using sub-brands to remain in stadiums after a sponsorship ban (23, 24). Fans saw sub-brands as a deliberate attempt to exploit legal loopholes, sometimes viewing this as “even more inappropriate than parent brand sponsorship” (24). This sense of betrayal is significant, as it links the ethical violation of circumventing public health laws to fans’ loyalty and trust in their clubs. Clubs accepting sponsorship from sub-brands clearly linked to gambling companies are seen as complicit in deception, damaging their integrity. Fans who felt betrayed were also most likely to support severe “sportive sanctions” against offending clubs, such as point deductions, relegation, or spectator bans (23). This shows that fans are active moral agents who judge club behavior and demand accountability.

However, not all fans viewed this circumvention the same way. A minority adopted a pragmatic perspective, recognizing the “financial motivations behind these strategies” and interpreting sub-branding as a “first step toward reduced visibility” (23, 24). This division reflects the broader tension between financial needs and ethical integrity seen in community sport studies. While most fans oppose gambling sponsorship, financial considerations and perceptions of sincerity influence the strength of this consensus. The presence of this dissenting minority also allows the gambling industry to frame its actions as a legitimate business response to restrictive regulation.

For sports organizations, integrity has become a key factor in shaping policy. Research on clubs in Belgium and the Netherlands (36) highlights that “integrity is a key factor shaping gambling-related policy and practice at the organisational level.” This indicates that accepting gambling sponsorship is increasingly seen as a decision affecting a club's reputation and moral standing, not just its finances. Clubs that partner with gambling companies may face backlash from fans, media scrutiny, and internal pressure. The focus on integrity serves as a strong internal motivator for change, separate from legal requirements. However, the same study calls for a “critical research agenda that can support the denormalisation of gambling” (36), noting that integrity-based arguments still compete with strong financial incentives and the widespread view of sports betting as a harmless social activity (36).

The emotional impact of gambling advertising on fans adds another ethical concern. A qualitative study with adult male football fans in the UK (37) explored the “immediate and ongoing emotions that football fans have or may not have towards gambling advertising.” While the abstract does not detail specific emotions, the study's focus suggests the issue is deeply affective, not just cognitive. In professional football, where gambling advertising is “almost unavoidable,” fans may feel annoyance, anxiety, or exploitation, especially if they have experienced gambling harm. This emotional aspect is crucial for understanding the full ethical cost of gambling sponsorship, as it affects fans’ well-being and enjoyment of sport.

To provide a structured synthesis of the evidence on stakeholder perceptions and ethical considerations, Supplementary Table S4 summarizes the key findings from the included studies.

An experimental study with cycling fans (38) offers insight into how sponsorship can shape ethical perceptions. The study found that when a gambling brand sponsors a road cycling team, fans see the brand as more altruistic than when it uses direct branded content. This perception reduces fans’ “inferences of manipulative intent,” which in turn increases both gambling urge and the company's reputation (38). This is ethically concerning, as sponsorship acts as a “Trojan horse,” associating a harmful brand with positive qualities like teamwork and achievement, and weakening fans’ critical resistance. The perceived altruism is a marketing illusion, yet it is highly effective, especially among vulnerable groups such as “young males,” for whom branded content directly improved corporate reputation (38). The study shows that the ethical issue with gambling sponsorship lies not only in promoting a harmful product, but also in how it strategically reshapes fans’ moral perspectives, turning critical viewers into supporters.

## 4 Discussion

The analysis of 29 studies reveals that gambling sponsorship is a deeply entrenched marketing practice, resistant to change but responsive to regulatory stringency. The literature consistently highlights the tension between the financial needs of sporting organizations and the public health goal of reducing gambling-related harm. This conflict is evident in the daily decisions of club administrators, fan reactions, and gambling operators’ strategies. The evidence indicates that comprehensive, statutorily enforced bans, such as the Spanish model (13, 26), are more effective in reducing gambling sponsorship than partial or voluntary industry-led measures, which are susceptible to displacement and circumvention (27–29).

Findings on gambling marketing exposure, content, and prevalence reveal widespread and increasing saturation. Longitudinal data from football shirt sponsorships in ten major leagues show a near tenfold rise in gambling sponsors from 2000 to 2022 (14), highlighting the industry's strategic focus on popular sports. This trend extends to esports (15), where gambling sponsorship targets younger, digitally native audiences. Marketing content is dominated by financial inducements, such as sign-up offers and bonus bets, especially during major tournaments (18, 19).

Experimental evidence shows these inducements strongly influence young people, increasing betting behavior and higher-risk gambling (21). Celebrity endorsements further enhance trust and reduce perceived risk (12). Multiple studies report high advertising recall among young people and higher-risk gamblers (20), indicating that marketing effectively reaches vulnerable groups. On social media, much content marketing breaches advertising standards by concealing its promotional nature (22), exposing a significant regulatory gap. This multi-channel saturation, combined with inducements and celebrity endorsements, normalizes gambling and is difficult to counter with public health messaging alone.

The evidence establishes a clear hierarchy of regulatory effectiveness. Comprehensive statutory bans, such as the Spanish Royal Decree 958/2020, significantly reduce marketing expenditure and key gambling behavior metrics, including new account registrations and total money wagered (13, 26). This shows that clear legal prohibitions with enforcement can disrupt the link between marketing and consumer behavior. However, these bans did not significantly change the behavior of existing active gamblers, and the use of bonuses as a marketing channel intensified (13, 26).

This highlights the need for regulations that cover all promotional activities, including bonuses and inducements, to prevent marketing from shifting to unregulated channels. In contrast, the UK's voluntary “whistle-to-whistle” ban has shown limited and inconsistent effectiveness. While advertising during restricted periods of live football broadcasts decreased, it was displaced to unrestricted periods and other sports (27–29). The continued presence of an average of three gambling advertisements per program (28) and increased gambling among higher-risk groups exposed to advertising (29) indicate that voluntary, partial restrictions are inadequate. The industry's ability to adapt and exploit loopholes demonstrates that self-regulation is ineffective when financial interests are at stake.

Regulatory circumvention through sub-branding, as seen in Belgium (23, 24), poses a significant challenge even to comprehensive statutory bans. Fans can easily recognize sub-brands and link them to their gambling parent brands, often expressing frustration and betrayal. This suggests that banning specific brand names is insufficient if operators can introduce related sub-brands that maintain consumer associations. Regulations must therefore be broad enough to include both primary gambling brands and any derivative or proxy brands. Strong fan support for punitive sanctions against clubs accepting such sponsorships (23, 24) indicates public demand for holding both the sponsored entity and the sponsor accountable. This shifts enforcement focus to sporting organizations, creating a strong deterrent by threatening outcomes most valuable to clubs, such as points, league position, and spectator support.

Stakeholder perceptions and ethical considerations provide important context for policy discussions. Multiple qualitative studies find that stakeholders see a fundamental moral conflict between the values of sport and the harms of gambling (33, 34). This moral judgment actively influences consumer behavior, with evidence showing that perceived moral inappropriateness harms both the sponsoring brand and the sponsored team (33). Public opinion can therefore support stricter regulation. However, financial pressures, especially at the community level, complicate this consensus. In Australia, wealthier clubs can refuse gambling sponsorship on ethical grounds, while poorer clubs—often in areas with higher gambling harm—are more likely to accept it (31, 35). This highlights a key policy implication: banning gambling sponsorship is necessary but not sufficient. Alternative, non-harmful funding sources must be provided to prevent the financial burden from falling on disadvantaged organizations. The view of gambling sponsorship as a “Trojan horse” that associates a harmful brand with sport (38) further supports the need for a complete separation between the two industries.

Furthermore, while the current literature predominantly focuses on elite men's sports—reflecting the highest concentrations of gambling sponsorship—there is a critical need to consider the impact on women's sports and lower-tier competitions. These leagues often operate with less diversified revenue streams, making them disproportionately dependent on sponsorship income (39, 40). Consequently, they are uniquely vulnerable to the financial shocks associated with the withdrawal of gambling sponsors. As elite leagues transition away from these partnerships due to public pressure or regulatory mandates, gambling operators may aggressively redirect their marketing investments toward these under-resourced segments. Policymakers must anticipate this displacement and ensure that alternative funding mechanisms are designed to protect the financial viability of women's and grassroots sports.

This synthesis has significant theoretical implications. The consistent effectiveness of comprehensive statutory bans over partial or voluntary measures supports a deterrence-based regulatory model, where clear rules and strong enforcement are needed to change industry behavior. However, evidence of displacement and circumvention shows the limits of reactive regulation. The gambling industry adapts quickly, so regulations must be dynamic and principle-based, focusing on the intent and effect of marketing rather than a fixed list of prohibited practices. For example, prohibiting any sponsorship that creates a cognitive association with a gambling brand, regardless of name or logo, is more robust than banning specific company names. Ethical literature further shows that the harm of gambling sponsorship extends beyond increased gambling behavior. It also erodes trust in sporting institutions, normalizes a harmful product, and imposes a moral burden on fans and community leaders who must balance financial needs with ethical integrity.

The practical implications for policymakers are clear. First, evidence supports comprehensive statutory bans on all forms of gambling sponsorship in sport, including shirt sponsorships, stadium naming rights, pitch-side advertising, and digital marketing. Partial bans, such as the UK's “whistle-to-whistle” model, are insufficient and can cause displacement effects. Second, bans must be broad enough to include sub-brands and proxy brands to prevent circumvention through rebranding. Third, enforcement must be robust, with meaningful sanctions for non-compliance applied to both gambling operators and sporting organizations. Strong fan support for sanctions like point deductions and relegation (23, 24) provides a public mandate. Fourth, policymakers should address the financial gap created by a ban, especially for community organizations, by providing alternative, non-harmful funding sources such as increased public investment, tax incentives for healthy sponsorships, or a levy on gambling operators for a central sports development fund. Finally, regulations should restrict the content of any remaining permissible marketing, such as banning sign-up offers and bonus bets, given their strong influence on young people.

This review has several limitations that should be considered when interpreting the findings. First, reliance on English-language publications may have excluded relevant research from non-English-speaking regions, particularly in Asia, South America, and parts of Europe. This language bias may limit the global applicability of the findings. Second, the focus on peer-reviewed academic literature and official reports excluded grey literature, such as industry reports and advocacy documents, which could provide additional insights into operator behavior and compliance. Including these sources might have offered a more complete picture of industry adaptation. Third, the diversity of study designs, outcome measures, and regulatory contexts among the 29 studies prevented a formal meta-analysis. While the narrative synthesis identified key patterns, it cannot provide a single, pooled estimate of the effect of sponsorship bans.

The varying methodological quality, especially between quasi-experimental and descriptive studies, also affects the strength of evidence across themes. Fourth, the recent publication window (2022 onwards) was chosen to reflect current regulatory environments but may have excluded important historical studies that offer valuable longitudinal context. Finally, publication bias is possible, as studies showing null or negative effects may be less likely to be published, potentially skewing the evidence toward a more optimistic view of regulatory effectiveness.

The gaps and inconsistencies identified in this review highlight key areas for future research. Longitudinal evaluations of complete sponsorship bans in jurisdictions like Belgium and the Netherlands are needed to assess changes in marketing exposure, gambling behavior, financial impacts on sporting organizations, and the emergence of circumvention tactics. Such studies would provide robust evidence on the long-term effectiveness of comprehensive bans. Future research should also examine specific circumvention tactics, including sub-branding (23, 24), affiliate marketing, influencer partnerships, and sponsorship of individual athletes or events.

Another understudied area is the impact of sponsorship bans on existing high-risk gamblers. Spanish evidence suggests bans are more effective at preventing new gamblers than changing established behaviors (13); further research should determine if this holds across contexts and what additional interventions may support harm reduction.

Moreover, the current evidence base exhibits a pronounced geographical bias toward European, Australian, and North American contexts, which inherently reflects the state of the available literature. However, this Western-centric focus limits the global applicability of the findings. Future research and policy discussions must acknowledge the rapidly evolving regulatory landscapes in emerging markets, particularly across the African continent, which is currently experiencing a massive gambling boom driven by mobile internet penetration and digital payment platforms. In these regions, where sports betting is growing exponentially and regulatory frameworks are often nascent, the vulnerability of populations to gambling-related harm is magnified. Understanding how cultural nuances, legal infrastructure, and economic dependencies affect the implementation and effectiveness of sponsorship restrictions in these rapidly expanding markets is crucial for developing robust global public health strategies.

Finally, the ethical and perceptual dimensions of gambling sponsorship require further exploration through large-scale quantitative surveys to measure ethical attitudes among fans and the public, and to assess how these attitudes are shaped by different sponsorship types and regulatory environments. This research could help policymakers gauge public support for various regulatory approaches and tailor communication strategies accordingly.

## 5 Conclusions

This systematic review analyzed 29 studies to assess how gambling bans and betting restrictions affect sports sponsorship, regulatory compliance, and consumer behavior in various jurisdictions. The findings show that gambling sponsorship is deeply rooted in elite sports, especially football, and has grown significantly over the past two decades. Comprehensive statutory bans, such as those in Spain, are more effective at reducing marketing exposure and gambling initiation than partial or voluntary self-regulation, which often leads to displacement and circumvention through sub-branding. The evidence also indicates that stakeholders recognize a moral conflict between sporting values and gambling promotion, but financial pressures, particularly at the community level, often hinder ethical decision-making.

This synthesis offers clear guidance for policymakers. Comprehensive bans on all forms of gambling sponsorship, backed by strong enforcement and meaningful penalties for both operators and sporting organizations, are the most effective regulatory strategy. These bans should be broad enough to include sub-brands and proxy marketing used to bypass restrictions. Regulatory reforms should also provide alternative funding for community sports to ensure that ethical decisions do not financially disadvantage the most vulnerable organizations. Theoretically, this review shows that gambling sponsorship harms not only through direct behavioral effects but also by eroding trust in sporting institutions and normalizing a harmful product through marketing associations.

Future research should focus on longitudinal studies of complete sponsorship bans in newly regulated jurisdictions, especially Belgium and the Netherlands, to assess long-term effectiveness and identify new circumvention tactics. There is also a need for studies on regulatory impacts in non-Western contexts, where sports betting is growing rapidly. Additionally, larger-scale quantitative research on stakeholder ethical attitudes would help policymakers gauge public support for various regulatory approaches and refine their communication strategies.

## Data Availability

The original contributions presented in the study are included in the article/Supplementary Material, further inquiries can be directed to the corresponding author/s.
