# Supplementary material for: Sports sponsorship, gambling bans, and regulatory compliance: a systematic review
Source: Front Sports Act Living. 2026 Aug 6;8:1900549. doi: 10.3389/fspor.2026.1900549 (PMC13490093; doi:10.3389/fspor.2026.1900549)
Supplement: Supplementary file 1 [file Supplementaryfile1.pdf]

Supplementary Material

1 Supplementary Figures

Figure 1. PRISMA flow diagram of the systematic literature search and study selection process

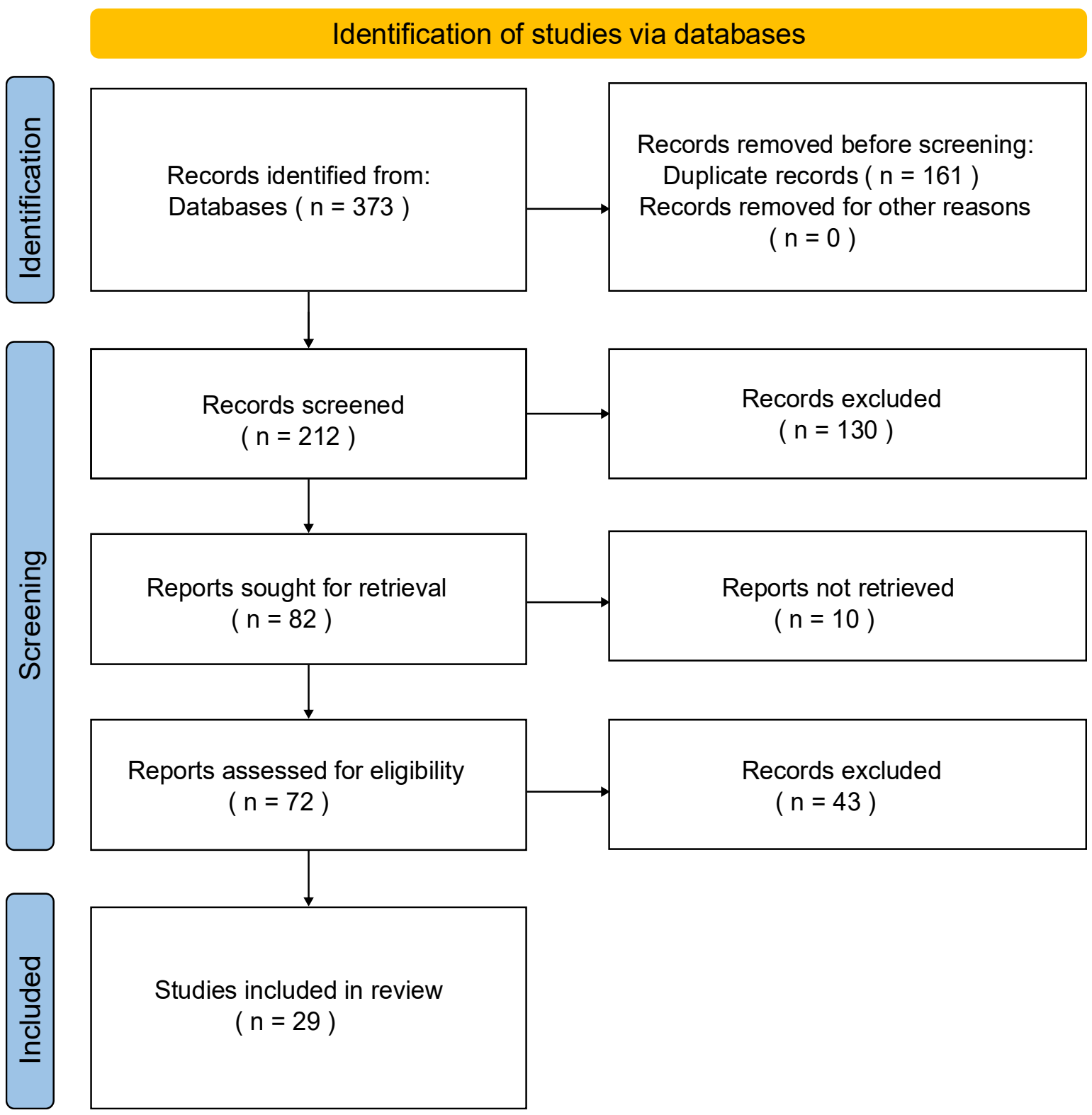

Figure 2. Research trends in the domain of sports sponsorship in relation to gambling bans or betting restrictions and regulatory compliance

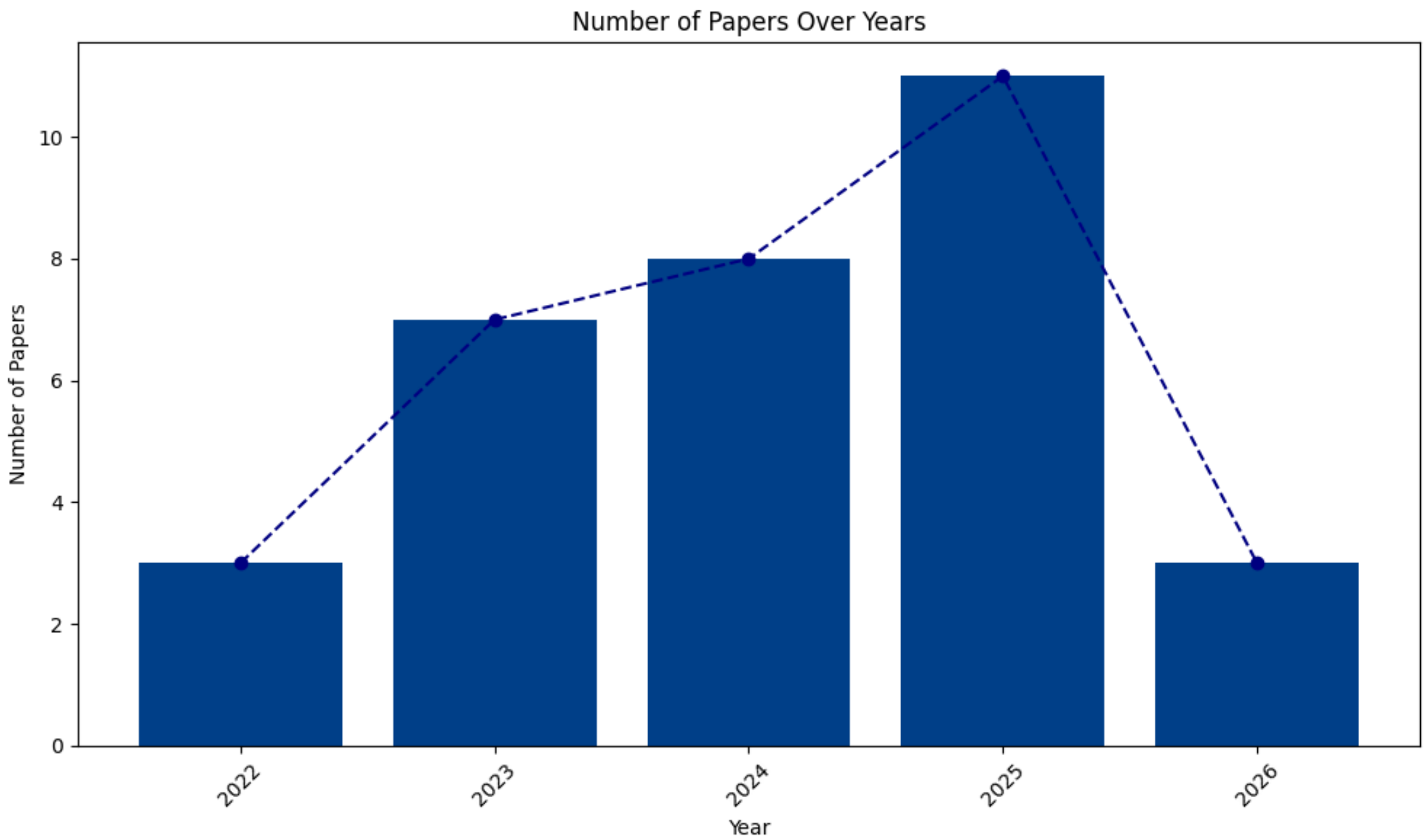

.

2 Supplementary Tables

Table 1. Characteristics of Included Studies

STUDY ID	STUDY DESIGN	SETTING	POPULATION	SPONSORSHIP OR ADVERTISING TYPE	REGULATORY CONTEXT	OUTCOMES MEASURED	KEY FINDINGS
(12)	Quasi-experimental using Seasonal Autoregressive Integrated Moving Average (SARIMA) models	Spain	Online gamblers (new accounts, active accounts, deposits, total money bet) and marketing expenditure data from the Directorate General for Regulation of Gambling	Advertising, bonuses, affiliation, sponsorship	Spanish Royal Decree 958/2020, which limits the marketing of gambling products, implemented between November 2020 and August 2021	New accounts, active accounts, deposits, total money bet, advertising expenditure, bonuses expenditure, affiliation expenditure, sponsorship expenditure	The regulation led to a permanent decrease in new accounts (-263k; p=0.003) and total money bet (-€216M; p=0.034), and reduced marketing expenditure on advertising (-€20M; p=0.004), bonuses (-€2.6M; p=0.048), and sponsorship (-€5.3M; p<0.001), but had little effect on active accounts.
(13)	Systematic review	Online data scraping of top esports teams competing in major tournaments for Dota 2, League of Legends (LoL), and CS:GO between October and November 2021	Top 20 esports clubs across Dota 2, League of Legends (LoL), and CS:GO	Gambling sponsorship		Level of gambling sponsorship of top esports teams; combined social media followers of teams sponsored by gambling companies	Half of the esports teams in Dota 2 and CS:GO's world championship events in 2021 were sponsored by gambling companies. Teams sponsored by gambling companies have a combined total of 25,868,912 followers across three major social media channels. No LoL teams were sponsored by gambling companies, yet sibling teams within the CS:GO and Dota 2 competitions were.
(14)	Qualitative focus group study	Inner and outer suburbs of Sydney, Australia	7–8 frequent sport spectators per group, including parents and non-parents	Unhealthy commodities sponsorship (unhealthy food, alcohol, and gambling products) in elite sport	Public health concerns in Australia about marketing of unhealthy commodities, particularly to children	Adults' perceptions of unhealthy commodities sponsorship in elite sport and policies to restrict them	Participants identified contradictions between healthy sport messages and unhealthy commodities, expressed concern about children's exposure to integrated marketing, supported restrictions based on perceived product harm (gambling seen as most harmful), supported policies reducing children's exposure but worried about financial risks to clubs, and identified governments and sports associations as responsible for change.
(15)	Mixed-methods analysis (content analysis and survey)	NFL broadcasts	College students (224 survey respondents)	Celebrity endorsements and promotional inducements in sports betting advertising	Post-2018 legalization wave	Perceived trustworthiness of betting operators, risk perception	Celebrity appearances significantly enhanced betting operators' perceived trustworthiness and reduced risk perception, outperforming monetary inducements; responsible gambling information had minimal visibility
(16)	Cross-sectional survey	United Kingdom, during the 2022 FIFA World Cup	190 UK residents aged 18–24 who had watched at least one 2022 World Cup match	Sports betting advertising across several media types, including social media, internet banners, and television; advertising for various bet types and betting inducements (e.g., in-play betting, sign-up offers,		Recall of sports betting advertisements across media types, recall of different bets and betting offers, problem gambling scores	Young people recalled a high amount of advertising for various bet types (95.6%) and betting inducements (89.5%); 64.2% recalled in-play betting ads and 68.1% recalled sign-up offers; higher-risk gamblers recalled more advertising than lower-risk gamblers; most ads recalled on social media (10-14 ads per week), then internet banners and television (5-9 ads per week each); only 46.3%

STUDY ID	STUDY DESIGN	SETTING	POPULATION	SPONSORSHIP OR ADVERTISING TYPE	REGULATORY CONTEXT	OUTCOMES MEASURED	KEY FINDINGS
				responsible gambling tools)			were aware of responsible gambling tool advertising.
(17)	Online survey		130 participants (71.5% male) aged between 18 and 24	Sports betting inducements (sign-up, bonus bets, increased odds, stake-back offers) resembling social media betting advertisements		Likelihood to place a bet, likelihood to engage in higher-risk betting, perceived value of each inducement	Sign-up and bonus bet inducements were perceived to have a stronger influence on increasing betting behaviour and engaging in higher-risk gambling compared to stake-back and increased odds inducements. These inducements were also seen as having greater promotional value. Those who experience gambling problems were more inclined to believe that incentives could motivate them to engage in riskier gambling behaviours.
(18)	descriptive study	live televised male sporting events broadcast in Ireland		gambling advertisements, including in-game fixed (static advertising) and dynamic (electronic advertisements changing at regular intervals) pitch-side advertising	before the adult television watershed in Ireland	frequency of gambling advertisements, type of gambling advertisements, evidence of responsible gambling (RG) practices (RG messaging, age limit, RG organization, responsible gambling tools)	Gambling advertisements were shown in 75.4% of games and were the seventh most commonly televised advertisement overall. Gambling advertising was more common in football compared to rugby and GAA. The majority of advertisements contained RG messaging, an age limit, and an RG organization. No advertisements showing responsible gambling tools were observed.
(19)	Online survey	Belgium, during the UEFA European Championships soccer (EURO 2024)	Belgian soccer fans, 67% male, average age 41.53 years (SD=17.4), total 1,611 records	Gambling marketing (including gambling sport sponsorship)	Extensive Belgian gambling marketing ban	Exposure to gambling marketing, recall of gambling marketing, normative perceptions, illusion of control, sports betting behaviors (EURO 2024 betting)	Direct effect between gambling marketing exposure and EURO 2024 betting (B=.98, SE=.11, 95%-CI=(.77; 1.19), p<.01). Higher marketing exposure led to higher normative perceptions (B=.63, SE=.03, 95%-CI=(.57; .70), p<.01), which increased likelihood of betting (B=.75, SE=.09, 95%-CI=(.58; .94), p<.01). Marketing exposure also had an effect on illusion of control (B=.36, SE=.03, 95%-CI=(.31; .41), p<.01), which had an effect on betting (B=.26, SE=.11, 95%-CI=(.05; .46), p<.01).
(20)		Belgium and the Netherlands	sports clubs	gambling industry sponsorship	deregulation	gambling-related policy and practice at the organisational level	integrity is a key factor shaping gambling-related policy and practice at the organisational level
(21)	Comparative analysis between two previous studies			Sponsorship of sports entities by gambling organizations (e.g., MGM Resorts) vs. non-gambling entities (e.g., Coca-Cola, Citibank)	U.S. Supreme Court ruling declaring the Professional and Amateur Sports Protection Act (PASPA) unconstitutional, allowing gambling organizations to sponsor sports entities	Congruence of benefits between sponsorship by gambling organizations and non-gambling entities, as derived from two studies documenting benefits to four categories of beneficiaries	
(22)	Longitudinal study	Spain		advertising, bonuses, affiliate marketing, sponsorship	Spanish Royal Decree 958/2020	new accounts, active accounts, gambler deposits, total money bet	Investment in advertising, promotions, and sponsorships significantly increase the number of new and active accounts, deposits, and total

STUDY ID	STUDY DESIGN	SETTING	POPULATION	SPONSORSHIP OR ADVERTISING TYPE	REGULATORY CONTEXT	OUTCOMES MEASURED	KEY FINDINGS
							money bet. For every €1 invested in bonuses and sponsorship, gamblers deposit €1.6 and €4 into their accounts, respectively. The Spanish law regulating gambling advertising weakened the link between marketing expenditure and gambling behavior, except for bonuses where the impact intensified.
(23)	Qualitative, mono method approach of semi-structured interviews with a small sample size, using thematic analysis and an abductive method	United Kingdom, within the context of professional football	Adult male football fans	Gambling advertising within or centred around professional football, including sponsorships and mass media advertising	Gambling Act of 2005, UK's Gambling Commission National Strategy to Reduce Gambling Harms (2019), safer gambling messages, gambling prevention and education	Immediate and ongoing emotions of football fans towards gambling advertising within professional football	
(24)	between-participants experiment	Instagram channel	181 cycling fans	gambling sponsorship (compared to branded content)		gambling urge, corporate reputation, perceived altruistic motives, inferences of manipulative intent	When a gambling brand sponsors a road cycling team, it is perceived as more altruistic, which decreases manipulative intent perceptions, which in turn increases gambling urge and corporate reputation. Branded content had a direct positive effect on corporate reputation only for young males.
(25)	Qualitative survey with open-ended questions	Belgium	200 sports fans	Gambling sub-brand sponsorship in sport	Extensive national regulations limiting gambling sponsorship since January 2025, with gambling companies using sub-brands to circumvent restrictions	Belgian football fans' ability to recall gambling sub-brands and their (non)associations with parent brands, and their moral evaluations of these sub-brands	Participants most often recalled gambling sub-brands and associated them with parent brands through semantic and categorical cues. Many fans expressed frustration and a sense of betrayal, believing sub-brands exploit legal loopholes. Other fans understood financial motivations and viewed subtle references as a step toward reduced visibility. Participants broadly supported sportive sanctions targeting the sponsored entity as the most effective deterrent.
(26)	qualitative survey	Belgium	Belgian football fans who watched at least one match per week	gambling sub-brands in sport sponsorship	extensive national regulations limiting gambling sponsorship in Belgium implemented in January 2025, with gambling companies using sub-brands to circumvent restrictions	football fans' ability to recall gambling sub-brands and their (non)associations with parent brands, and their moral evaluations of gambling sub-brands	Participants most often recalled gambling sub-brands and associated them with parent brands through semantic cues (color, typography, logo, visual style) and categorical cues (references to sports or gambling). Many fans expressed frustration and a sense of betrayal, viewing sub-brands as even more inappropriate than parent brand sponsorship. Other fans understood financial motivations and saw sub-brands as a step toward reduced visibility. Participants broadly supported sportive sanctions such as point deductions, relegation, and spectator bans.

STUDY ID	STUDY DESIGN	SETTING	POPULATION	SPONSORSHIP OR ADVERTISING TYPE	REGULATORY CONTEXT	OUTCOMES MEASURED	KEY FINDINGS
(27)	Audit	Western Australia	Local Government Authorities (LGAs) (n=139)	Sponsorship, signage, venue hire, and community grants related to harmful commodities (alcohol, tobacco, gambling products, unhealthy food and beverages)	Local government sponsorship and signage policies	Inclusion of statements restricting the promotion of harmful commodities via sponsorships, signage, venue hire, and sporting and/or community grants policies	Six percent (n=28) of policies included statements restricting the promotion of at least one harmful commodity. Twenty-three local governments had at least one policy that restricted unhealthy signage or sponsorship. Most WA local governments do not have publicly available policies that specifically limit the advertising and promotion of harmful commodities in their government-owned facilities.
(28)	Two single-factor between-subjects experiments		200 adult participants per experiment	Gambling company sports sponsorship vs. banking company sports sponsorship		Gambling-linked outcomes, team outcomes, brand outcomes, sponsor moral appropriateness, normalization and risk perceptions about gambling	Participants did not see a good fit between a commercial gambling provider and a soccer team, perceived gambling sponsorship as morally inappropriate (among people who did not experience any risks or problems with gambling), and found the gambling sponsor insincere. Gambling (vs. banking) sports sponsorships directly negatively influenced the sponsoring brand and sponsored team. Gambling-linked outcomes were indirectly negatively affected by gambling sponsorships via sponsor moral appropriateness and normalization and risk perceptions about gambling.
(29)	Retrospective study	First-division football teams in 10 countries (Argentina, Brazil, England, France, Germany, Italy, Poland, the Netherlands, Spain, and the United States) from 2000–2022	First-division football teams in the top ten football leagues	Alcohol, unhealthy food (ultra-processed food according to NOVA classification), gambling, other, and tobacco		Proportion of sponsors on team jerseys categorized by type (alcohol, unhealthy food, gambling, other)	Majority of sponsorships were 'other' (81.8%), followed by gambling (6.9%), alcohol (2.6%), and unhealthy food (2.6%). No tobacco sponsors identified. Gambling sponsors increased from 1.7% in 2000 to 16.3% in 2022; alcohol sponsors decreased from 6.2% to 1.0%. Trends were statistically significant. Alcohol industry remained visible in the Spanish league.
(30)	Content analysis	Czech Republic, Czech First Football League broadcasts	32 matches during August–September 2025	Gambling advertisements (sports betting, online casino, lottery)	Czech Republic	Frequency, duration, timing, product types, and promotional characteristics of gambling advertisements	A total of 1,824 gambling advertisements were documented across 32 matches, averaging 57 advertisements per match (SD = 7.04). Total advertising duration was 22,011 seconds (6.1 hours), with an average of 11.5 minutes per match. Sports betting dominated (85.0%), followed by online casino promotions (10.7%) and lottery advertisements (4.3%). During live match coverage, 700 advertisements from a single title sponsor were recorded.
(31)	Qualitative study using in-depth interviews and reflexive	Community sporting organizations in Australia	Senior leaders in community sporting organizations (n = 24)	Gambling sponsorship, including from community clubs	Global regulatory reform efforts addressing normalization of gambling;	Factors shaping decisions about accepting or refusing gambling	Participants considered alignment of values between sponsor and organization; some forms of sponsorship (e.g., from community clubs) were perceived as less harmful

STUDY ID	STUDY DESIGN	SETTING	POPULATION	SPONSORSHIP OR ADVERTISING TYPE	REGULATORY CONTEXT	OUTCOMES MEASURED	KEY FINDINGS
	thematic analysis			and hotels, and online wagering	Australian context with gambling companies and venues sponsoring community sport	sponsorships and partnerships	than others (e.g., online wagering); concerns about ethical engagement and overall community impact; decisions influenced by perceived risks and benefits, including impact on reputation and need for financial stability
(32)	Qualitative study using in-depth interviews	Australian community sporting organisations	24 key decision makers in Australian community sporting organisations	Gambling sponsorship	Unlike tobacco, there are no restrictions relating to gambling sponsorship in Australian sports	Attitudes towards gambling, decisions about gambling sponsorship within clubs, issues relating to alternative sponsors and sponsorship disparities	Sporting organisations navigate ethical dilemmas when considering gambling sponsorships, weighing values, reputation, and financial viability. Some would refuse gambling sponsorship for ethical reasons but recognised that poorer clubs may be more vulnerable. As elite sporting organisations move away from gambling sponsorship, gambling companies may target community sport.
(33)	Observational study using linear regression models	United Kingdom television		Gambling advertising	Voluntary partial gambling advertising restrictions known as the 'whistle-to-whistle' (W2W) ban	Changes in the frequency of gambling advertising during sports (football, horse racing, other) and other television programming (documentaries, drama, entertainment, film, leisure, music, news, other)	The W2W ban was associated with a decrease in gambling advertising around live football (2.9 advertisements per-program; $p < .001$) and other live sports (0.8 advertisements per-program; $p < .001$), except horse racing where advertising increased (2.5 advertisements per-program; $p < .001$). There were small changes in advertising around other types of programming during the same years.
(34)	Inverse Probability Weighted (IPW) matching models	United Kingdom, television broadcasts of live football games		Gambling advertising	Voluntary partial advertising ban during live sports broadcasts known as the 'whistle-to-whistle' ban, introduced in 2019	Frequency of television advertising around live football games, including pre-match, half-time, and overall per-programme advertising counts	The ban was associated with a reduction of 2.3 advertisements per-programme overall, driven by reductions during half-time (2.18 advertisements per-programme). There was an increase of 0.34 advertisements per-programme during the pre-match section. An average of 3 advertisements per-programme remained post-ban.
(35)	Systematic review and econometric methods using primary self-reported gambling data, and secondary television advertising and scheduling data; Quantitative analysis included a quasi-experiment during the 2022 football World Cup, and an econometric analysis of the impact of existing advertising	Television during live sports, with a particular focus on live football and horse racing broadcasts.	General population, with a focus on higher-risk gamblers.	Gambling advertising on television during live sports.	Industry-led advertising restriction known as the 'whistle-to-whistle' (W2W) ban introduced in 2019 in the UK; self-regulatory measures.	Overall frequency of bets placed, probability of placing a bet in a higher-risk population group, presence of advertising on television.	Existing evidence suggests sports-related gambling advertising is associated with increases in gambling behaviour. Self-reported effects of advertising appear higher in higher-risk gamblers. Advertising during live World Cup broadcasts influenced frequency of bets and probability of betting in a higher-risk group. The W2W ban reduced advertising during live football but was associated with an increase in advertising around live horse racing, with little impact on non-sports programming. The ban reduced total frequency of advertising during live sports, but advertising may have spread to unrestricted live-sports programming, raising concerns about overall efficacy of industry-led restrictions.

STUDY ID	STUDY DESIGN	SETTING	POPULATION	SPONSORSHIP OR ADVERTISING TYPE	REGULATORY CONTEXT	OUTCOMES MEASURED	KEY FINDINGS
	restrictions on the presence of advertising on television. Regression models included linear, count, Propensity Score (PSM), and Inverse Probability Weighted (IPW) matching models.						
(36)	Observational study	United Kingdom, during the men's 2020 Euro soccer tournament	Televised gambling advertisements aired during matches of the tournament	Television advertising of gambling	Voluntary 'whistle-to-whistle ban' on gambling advertising	Frequency and content of gambling adverts, categories of adverts (financial inducements, brand awareness, odds on complex bets, safer gambling), presence of safer gambling messages	113 gambling adverts were recorded (4.5 adverts per relevant match). Financial inducements were the most frequent category (56.6%), followed by brand awareness (19.5%), complex bet odds (18.6%), and safer gambling (5.3%). The 'when the fun stops, stop' message appeared in 56.6% of adverts.
(37)	Frequency analysis	Live televised coverage of Premier League fixtures in the UK, Sky Sports News broadcasts, TalkSport radio broadcasts, and social media platforms (Facebook, Instagram, Twitter/X)		Gambling marketing and gambling advertising	Industry self-regulation	Frequency of gambling marketing during live broadcasts, Sky Sports News broadcasts, TalkSport radio broadcasts, and on social media	
(38)	Observational study	Live TV coverage of the 2022 Qatar FIFA Men's World Cup on the commercial broadcaster ITV	Gambling adverts shown during ITV's live coverage of matches	Gambling adverts (financial inducements, live odds, safer gambling, brand awareness)	Gambling industry self-regulation such as the whistle-to-whistle ban	Prevalence and features of gambling adverts, including timing, advert category, and safer gambling messaging	Over 30 matches, there were 156 gambling adverts (M=5.2 per match, range 3-8). Majority shown pre-match (80.8%). Financial inducements most common (42.3%), followed by brand awareness (26.9%). Safer gambling message 'take time to think' appeared in 70.5% of adverts; lottery adverts had no safer gambling messages.
(39)		International high-level men's soccer leagues (English Premier League, Italy, Spain)		Gambling, cryptocurrency, and financial trading sponsorships	Premier League plans to ban gambling shirt-front sponsorships by 2026 (excluding shirt sleeves and pitch-side hoardings); Italy and Spain have fully banned gambling sponsorships and in-game marketing due		In the 2022/23 English Premier League season, eight clubs collectively received an estimated £60 million from gambling shirt-front sponsorships. Cryptocurrency and financial trading sponsorships are emerging as gambling-like products, engaged in disproportionately by those experiencing gambling-related harm, and use soccer to market themselves.

STUDY ID	STUDY DESIGN	SETTING	POPULATION	SPONSORSHIP OR ADVERTISING TYPE	REGULATORY CONTEXT	OUTCOMES MEASURED	KEY FINDINGS
					to public health concerns		
(40)	observational	televised sports matches broadcast in Ontario and social media (X/Twitter) advertisements by Ontario-licensed gambling operators	televised broadcasts of five NHL and two NBA matches; ten Ontario-licensed gambling operators' X/Twitter advertisements	gambling advertisements	Ontario-based advertising standards	prevalence of gambling messages, regulatory compliance, visibility duration, audience demographics, content marketing identification	4,119 gambling messages (3,537 television-based, 582 X/Twitter); 50.4% of TV references on playing surface; visible for 21.7% of broadcast duration; no TV breaches; X/Twitter ads generated 5,687,087 views, mostly males (78.5%) aged 25-34 (50.5%); 48.5% were content marketing breaching standards by concealing advertisement nature

Table 2. Summary of Studies on Exposure, Content, and Prevalence of Gambling Marketing in Sport

STUDY ID	GAMBLING MARKETING TYPE	MEDIA CHANNEL	EXPOSURE CONTEXT	REGULATORY COMPLIANCE ISSUES	KEY FINDINGS ON IMPACT
(25)	sub-brand sponsorship	sports media	sport	circumvention of restrictions through sub-brands operating in sectors such as sports media, exploiting legal loopholes	fans expressed frustration and a sense of betrayal, believing sub-brands deliberately exploit legal loopholes; other fans understood financial motivations and viewed subtle references as a first step toward reduced visibility; fans broadly supported sportive sanctions (point deductions, relegation, spectator bans, match-based fines) targeting the sponsored entity
(26)	sub-branding strategy	sports media	sport sponsorship, specifically Belgian football	circumvention of extensive national regulations limiting gambling sponsorship in Belgium through introduction of sub-brands; legal proceedings against the government	participants recalled gambling sub-brands and associated them with parent brands via semantic and categorical cues; many fans expressed frustration and a sense of betrayal, viewing sub-brands as even more inappropriate than parent brand sponsorship; some fans understood financial motivations and saw sub-brands as a step toward reduced visibility; participants broadly supported sportive sanctions such as point deductions, relegation, and spectator bans
(29)	primary sponsors on team jerseys	football club jerseys	top ten football leagues (Argentina, Brazil, England, France, Germany, Italy, Poland, the Netherlands, Spain, and the United States) from 2000–2022		The prevalence of gambling sponsors surged from 1.7% in 2000 to 16.3% in 2022; this trend was statistically significant.
(30)	Sports betting, online casino promotions, lottery advertisements	Television broadcasts of Czech football matches	During live match coverage of the Czech First Football League	High levels of gambling advertising exposure during sports broadcasts, suggesting need for regulatory consideration	Extensive exposure to gambling advertising during Czech football broadcasts, substantially exceeding levels documented in prior studies
(13)	sponsorship	social media channels	professional esports teams competing in major tournaments (Dota 2, League of Legends, CS:GO)		Half of the esports teams in Dota 2 and CS:GO's world championship events in 2021 were sponsored by gambling companies. Teams sponsored by gambling companies have a combined total of 25,868,912 followers across three major social media channels. No LoL teams were sponsored by gambling companies, yet sibling teams within the CS:GO and Dota 2 competitions were.
(35)	television gambling advertising	television	live sport, specifically live football broadcasts and live horse racing	industry-led 'whistle-to-whistle' (W2W) ban; concerns about overall efficacy of industry-led advertising restrictions; suggestion that UK gambling advertising policy may require stronger regulation beyond current self-regulatory measures	television advertising around live sports increases gambling behaviour amongst a high-risk group; the W2W ban reduced advertising frequency during live football but was associated with an increase in advertising around live horse racing not subject to restrictions; restrictions had little impact on non-sports programming
(36)	television advertising	television	men's 2020 Euro soccer tournament	industry's voluntary 'whistle-to-whistle ban' on gambling advertising	gambling advertising remains frequent (4.5 adverts per relevant match) during live televised soccer in the UK
(37)	Live Televised Coverage Analysis, Sky Sports News Analysis, TalkSport Radio Analysis, Social Media Analysis	Television (Premier League broadcasts, Sky Sports News), Radio (TalkSport), Social Media (Facebook, Instagram, Twitter/X)	Live broadcasts of Premier League fixtures, Sky Sports News broadcasts, TalkSport radio broadcasts, social media feeds	Industry self-regulation failure	
(38)	TV adverts for gambling brands or products, including	Commercial broadcaster	Live coverage of the 2022 Qatar FIFA	Gambling industry self-regulation such as whistle-to-whistle ban; adverts for lottery	Multiple gambling adverts shown during each match (mean 5.2, range 3-8), majority pre-match (80.8%), pre-match adverts predominantly

STUDY ID	GAMBLING MARKETING TYPE	MEDIA CHANNEL	EXPOSURE CONTEXT	REGULATORY COMPLIANCE ISSUES	KEY FINDINGS ON IMPACT
	financial inducements, live odds, safer gambling, and brand awareness	ITV's live TV coverage	Men's World Cup matches	products did not feature any safer gambling messages	encourage viewers to gamble promptly through financial inducements and boosted live odds
(39)	gambling sponsorships, cryptocurrency sponsorships, financial trading sponsorships	shirt-front sponsorships, shirt sleeves, pitch-side hoardings	in-game marketing in high-level men's soccer leagues	Premier League plans to ban gambling shirt-front sponsorships by 2026, but not shirt sleeves or pitch-side hoardings; Italy and Spain have fully banned gambling sponsorships and in-game marketing due to public health concerns	gambling sponsorships provide substantial funds to clubs; cryptocurrency and financial trading are gambling-like products engaged in disproportionately by those experiencing gambling-related harm and use soccer to market themselves
(40)	televised gambling references and social media advertisements	television and X/Twitter	during five NHL and two NBA matches broadcast in Ontario	none of the television-based references breached Ontario-based standards; content marketing on X/Twitter was found to be breaching advertising standards by concealing the fact that they were advertisements	high volume of marketing risks the possible normalization of gambling within sport
(15)	celebrity endorsements and promotional inducements	NFL broadcasts	sports betting advertising	minimal visibility of responsible gambling information	celebrity appearances significantly enhanced betting operators' perceived trustworthiness and reduced risk perception, outperforming monetary inducements
(16)	sports betting advertising, in-play betting, sign-up offers, betting inducements	social media, internet banners, television	during the 2022 FIFA World Cup	less than half of respondents were aware of advertising for responsible gambling tools; need for policy measures to limit young people's exposure to gambling advertising	young people recalled high amounts of advertising for various bets (95.6%) and inducements (89.5%); 64.2% recalled in-play betting, 68.1% recalled sign-up offers; higher-risk gamblers recalled more advertising; respondents recalled 10-14 ads per week on social media, 5-9 on internet banners and television
(17)	sports betting inducements	social media	participants were presented with four betting inducements that resembled social media betting advertisements	implementing policies to restrict inducements for sports betting could help mitigate gambling-related harm among young people	sign-up and bonus bet inducements were perceived to have a stronger influence on increasing betting behaviour and engaging in higher-risk gambling compared to stake-back and increased odds inducements; these inducements were also seen as having greater promotional value; those who experience gambling problems were more inclined to believe that incentives could motivate them to engage in riskier gambling behaviours
(18)	Television advertisements, static pitch-side advertising, dynamic pitch-side advertising	Television	During live televised male sporting events (Association Football, Rugby Union, Gaelic Athletic Association matches) in Ireland, including pre-match, half-time, and post-match periods	Gambling advertisements shown before the adult television watershed; no advertisements showing responsible gambling tools; gambling legislation required to minimize harm to vulnerable groups including children	Gambling advertisements were shown in 75.4% of games and were the seventh most commonly televised advertisement overall; gambling advertising was more common in football than in rugby or GAA; static and dynamic gambling advertising were common during football matches

Table 3. Summary of Studies on the Effectiveness and Impact of Gambling Advertising and Sponsorship Restrictions

STUDY ID	INTERVENTION OR RESTRICTION	REGULATORY TYPE	ANALYTICAL METHOD	BEHAVIORAL IMPACT	REGULATORY COMPLIANCE OUTCOME	SPONSORSHIP CONTEXT
(12)	Limits on marketing, bonuses, and sponsorship via Royal Decree 958/2020	Statutory, comprehensive national ban	Seasonal Autoregressive Integrated Moving Average (SARIMA) models	Permanent decrease in new accounts and total money bet; no significant impact on active accounts	The regulation led to a decrease in gambling behavior and marketing expenditure, indicating compliance with the Royal Decree	Online gambling in Spain
(22)	Marketing restrictions under the Spanish Royal Decree 958/2020	Statutory, comprehensive national ban	Seasonal Autoregressive Integrated Moving Average (SARIMA) model	The law weakened the link between marketing and gambling behavior for most channels, except for bonuses where the impact intensified.	Compliance was achieved, but adaptive marketing (bonuses) remained potent.	Online gambling in Spain
(33)	Voluntary 'whistle-to-whistle' (W2W) ban	Industry self-regulation, partial ban	Linear regression models	Decrease in advertising around live football and other live sports; increase around live horse racing.	Advertisements were not eliminated; displacement to horse racing occurred.	Live sports television in the UK
(34)	Voluntary 'whistle-to-whistle' (W2W) ban	Industry self-regulation, partial ban	Inverse Probability Weighted (IPW) matching models	Reduction in advertising during the restricted period (half-time); increase in advertising during unrestricted period (pre-match).	An average of 3 advertisements per-programme remained post-ban.	Live football television in the UK
(35)	Voluntary 'whistle-to-whistle' (W2W) ban; self-regulation	Industry self-regulation, partial ban	Systematic review, quasi-experiment, econometric analysis (PSM, IPW)	Advertising increased gambling behaviours in a high-risk group. The W2W ban reduced advertising during live football but increased it around horse racing.	Concerns about overall efficacy of industry-led restrictions were raised.	Live sports (football, horse racing) in the UK
(25)	Comprehensive national regulations limiting gambling sponsorship (sub-brand analysis)	Statutory, comprehensive national ban (with circumvention)	Qualitative survey	N/A (focus on fan recall and moral evaluation of circumvention)	Operators circumvented the ban using sub-brands. Fans expressed frustration and supported punitive sanctions for teams.	Belgian football sponsorship
(26)	Comprehensive national regulations limiting gambling sponsorship (sub-brand analysis)	Statutory, comprehensive national ban (with circumvention)	Qualitative survey	N/A (focus on fan recall and moral evaluation of circumvention)	Operators circumvented the ban by using sub-brands; this was perceived negatively by fans.	Belgian football sponsorship
(27)	Audit of local government policies restricting harmful commodities sponsorship	Local government policy	Policy audit	N/A (no behavioral data)	Most local governments lacked policies to limit harmful commodity promotion in their venues.	Community sports venues in Western Australia
(39)	Comparison of Premier League's partial ban vs. Italy and Spain's full bans	Statutory, comprehensive (Italy/Spain) vs. partial (EPL)	Descriptive, observational	N/A (no behavioral data)	Partial bans leave significant avenues for marketing; full bans are recognized as a stronger public health response.	High-level men's soccer leagues

Table 4. Summary of Studies on Stakeholder Perceptions and Ethical Considerations of Gambling Sponsorship

STUDY ID	STAKEHOLDER GROUP	METHODOLOGY	PERCEIVED MORAL APPROPRIATENESS	KEY ETHICAL CONCERN	ATTITUDE TOWARD FINANCIAL VS. ETHICAL TENSION	POLICY RECOMMENDATION OR SUPPORT
(14)	Australian sporting fans (parents and non-parents)	Four focus groups	Gambling viewed as having the greatest health impact among unhealthy commodities; significant moral dissonance with sport.	Children's exposure to integrated marketing that encourages unhealthy habits.	Supportive of policies reducing children's exposure, but concerned about financial risk to clubs.	Governments and sports associations responsible for enacting change.
(20)	Sports clubs in Belgium and the Netherlands	Empirical insights from two related studies	Not explicitly assessed.	Integrity as a key factor shaping policy; need to denormalize gambling.	Not explicitly assessed.	Support for denormalisation of gambling and deconstruction of "fun, risk-free" discourse.
(23)	Adult male football fans in the UK	Qualitative, semi-structured interviews	Not explicitly assessed.	The immediate and ongoing emotions caused by gambling advertising; lack of government action.	Discusses guidance to viewers and responsibility of organizations and government to protect the public.	Not specified in abstract.
(24)	Cycling fans	Between-participants experiment	Not explicitly assessed (focus on altruism perceptions).	Sponsorship is perceived as more altruistic, reducing manipulative intent inferences and increasing gambling urge and corporate reputation.	Not explicitly assessed.	Provides theoretical and practical insights into the impact on vulnerable groups (e.g., young males).
(25)	Belgian football fans	Qualitative survey with open-ended questions	Sub-brands viewed as deliberately exploiting legal loopholes; many fans expressed frustration and a sense of betrayal.	Gambling sub-brands circumventing regulations to maintain visibility.	Mixed: frustration vs. understanding of financial motivations; some saw sub-brands as a step toward reduced visibility.	Support for sportive sanctions (point deductions, relegation, spectator bans, match-based fines) targeting the sponsored entity.
(26)	Belgian football fans	Qualitative survey	Sub-brands perceived as even more inappropriate than parent brand sponsorship by many fans.	The moral evaluation of indirect and ambiguous sub-brands; perceived deliberate exploitation of legal loopholes.	Mixed: frustration vs. understanding of financial motivations.	Support for sportive sanctions (point deductions, relegation, spectator bans).
(28)	General consumers	Two single-factor between-subjects experiments	Gambling sponsorship perceived as morally inappropriate (among non-problem gamblers); sponsor seen as insincere.	Poor fit between gambling company and soccer team; moral inappropriateness leads to negative brand and team outcomes.	Not explicitly assessed.	Implies moral judgment is a protective mechanism.
(31)	Senior leaders in Australian community sport	In-depth interviews	Most stated that gambling and sport should not be aligned; some forms (community clubs) seen as less harmful than others (online wagering).	Alignment of values; ethical engagement with sponsors; overall community impact.	Decisions balanced perceived risks/benefits, reputation, and need for financial stability.	Regulation, formal policies, and education about risks.
(32)	Key decision-makers in Australian community sport	In-depth interviews	Some would refuse gambling sponsorship for ethical reasons.	Sporting organisations navigate ethical dilemmas weighing values, reputation, and financial viability.	Poorer clubs recognized as more vulnerable to accepting gambling sponsorship.	Policy action is needed to protect local communities.

Appendix: PRISMA 2020 Checklist

Section/Topic	Item #	Checklist item	Location in Manuscript
**TITLE**			
Title	1	Identify the report as a systematic review.	Title
**ABSTRACT**			
Abstract	2	See the PRISMA 2020 for Abstracts checklist.	Abstract
**INTRODUCTION**			
Rationale	3	Describe the rationale for the review in the context of existing knowledge.	Introduction
Objectives	4	Provide an explicit statement of the objective(s) or question(s) the review addresses.	Introduction
**METHODS**			
Eligibility criteria	5	Specify the inclusion and exclusion criteria for the review and how studies were grouped for the syntheses.	Methodology (Inclusion and Exclusion Criteria)
Information sources	6	Specify all databases, registers, websites, organisations, reference lists and other sources searched or consulted to identify studies.	Methodology (Review Protocol)
Search strategy	7	Present the exact search strategies used for all databases, registers and websites, including any filters and limits used.	Methodology (Review Protocol)
Selection process	8	Specify the methods used to decide whether a study met the inclusion criteria of the review, including how many reviewers screened each record and each report retrieved.	Methodology (Study Selection Process)
Data collection process	9	Specify the methods used to collect data from reports, including how many reviewers collected data from each report.	Methodology (Review Protocol / Analytical Dimensions)
Data items	10	List and define all outcomes for which data were sought. Specify all other variables for which data were sought.	Methodology (Analytical

Section/Topic	Item #	Checklist item	Location in Manuscript
			Dimensions for Synthesis)
Study risk of bias assessment	11	Specify the methods used to assess risk of bias in the included studies, including details of the tool(s) used, how many reviewers assessed each study and whether they worked independently.	Methodology (Quality Appraisal and Risk of Bias)*
Effect measures	12	Specify for each outcome the effect measure(s) (e.g. risk ratio, mean difference) used in the synthesis or presentation of results.	Results (Overview of Included Studies)
Synthesis methods	13	Describe the processes used to decide which studies were eligible for each synthesis. Describe any methods required to prepare the data for synthesis.	Methodology (Analytical Dimensions for Synthesis)
Reporting bias assessment	14	Describe any methods used to assess risk of bias due to missing results in a synthesis (arising from reporting biases).	Discussion (Limitations paragraph)
Certainty assessment	15	Describe any methods used to assess certainty (or confidence) in the body of evidence for an outcome.	Discussion (Limitations paragraph)
**RESULTS**			
Study selection	16	Describe the results of the search and selection process, from the number of records identified in the search to the number of studies included in the review, ideally using a flow diagram.	Methodology (Study Selection Process) & Figure 1
Study characteristics	17	Cite each included study and present its characteristics.	Results (Table 1: Characteristics of Included Studies)
Risk of bias in studies	18	Present assessments of risk of bias for each included study.	Methodology (Quality Appraisal and Risk of Bias)*
Results of individual studies	19	For all outcomes, present, for each study: (a) summary statistics for each group (where appropriate) and (b) an effect estimate and its precision (e.g. confidence/credible interval), ideally using structured tables or plots.	Results (Tables 2, 3, and 4)

Section/Topic	Item #	Checklist item	Location in Manuscript
Results of syntheses	20	For each synthesis, briefly summarise the characteristics and risk of bias among contributing studies. Present results of all statistical syntheses conducted.	Results (Thematic subsections)
Reporting biases	21	Present assessments of risk of bias due to missing results (arising from reporting biases) for each synthesis assessed.	Discussion (Limitations paragraph)
Certainty of evidence	22	Present assessments of certainty (or confidence) in the body of evidence for each outcome assessed.	Discussion (Limitations paragraph)
**DISCUSSION**			
Discussion	23	Provide a general interpretation of the results in the context of other evidence. Discuss any limitations of the evidence included in the review. Discuss any limitations of the review processes used. Discuss implications of the results for practice, policy, and future research.	Discussion & Conclusions
**OTHER INFORMATION**			
Registration and protocol	24	Provide registration information for the review, including register name and registration number, or state that the review was not registered.	Methodology (Review Protocol)
Support	25	Describe sources of financial or non-financial support for the review, and the role of the funders or sponsors in the review.	Funding
Competing interests	26	Declare any competing interests of review authors.	Conflict of Interest
Availability of data, code and other materials	27	Report which of the following are publicly available and where they can be found: template data collection forms; data extracted from included studies; data used for all analyses; analytic code; any other materials used in the review.	Data Availability Statement
